# Combined use of intravenous and topical versus intravenous tranexamic acid in primary total knee and hip arthroplasty: a meta-analysis of randomised controlled trials

**DOI:** 10.1186/s13018-017-0520-4

**Published:** 2017-02-02

**Authors:** Jun-feng Li, Hang Li, Hui Zhao, Jun Wang, Shen Liu, Yang Song, Hong-fen Wu

**Affiliations:** 1 Ji Lin Agricultural University Hospital, Ji Lin, China; 2 Chest surgery, Cancer hospital of Ji Lin, Ji Lin, China; 3 Integrated TCM & Western Medicine Department, Cancer hospital of Ji Lin, Ji Lin, China; 4 Pharmacy Department, Cancer hospital of Ji Lin, Ji Lin, China; 5 Department of Radiotherapy, Cancer hospital of Ji Lin, Changchun, Ji Lin China

**Keywords:** Total hip replacement, Total knee replacement, Tranexamic acid, Meta-analysis, Total knee and hip arthroplasty, Blood loss

## Abstract

**Background:**

This meta-analysis aimed to evaluate the efficiency and safety of combined intravenous and topical methods of application versus single intravenous of tranexamic acid in primary total knee and hip arthroplasty.

**Methods:**

A systematic search was carried out in MEDLINE (from 1966 to 25 September 2016), PubMed (from 1966 to 25 September 2016), Embase (from 1980 to 25 September 2016), ScienceDirect (from 1985 to 25 September 2016) and the Cochrane Library. Only high-quality randomised controlled trials (RCT) were identified. Two authors independently performed data extraction and quality assessment of included studies. Meta-analysis was conducted using Review Manager 5.1 software.

**Results:**

Six RCTs that included 687 patients met the inclusion criteria. The present meta-analysis indicated that there were significant differences in terms of total blood loss (MD = −193.59, 95% CI −338.06 to −49.13, *P* = 0.009), transfusion rate (RD = −0.07, 95% CI −0.12 to −0.03, *P* = 0.001), haemoglobin decline (MD = −0.51, 95% CI −0.83 to −0.18, *P* = 0.01) and length of stay (MD = −0.20, 95% CI −0.38 to −0.02, *P* = 0.03) between groups.

**Conclusions:**

Combined administration of tranexamic acid (TXA) in patients with total knee and hip arthroplasty was associated with significantly reduced total blood loss, transfusion requirements, postoperative haemoglobin decline and length of stay compared to single application alone but was not associated with prolonged operation time. Moreover, no adverse effects, such as superficial infection, deep vein thrombus (DVT) or pulmonary embolism (PE), were associated with TXA. We suggest that combined administration of TXA demonstrated excellent clinical efficacy and safety in patients with total knee and hip arthroplasty. More importantly, well-designed studies with larger sample size are needed to provide further reliable evidence for the combined use of TXA.

## Background

Total knee arthroplasty and hip arthroplasty (TKA and THA) are the most commonly employed orthopaedic operations to treat degenerative arthritis and traumatic conditions such as displaced femoral neck fractures. It has been estimated that more than 700,000 TKAs are performed annually in the USA. However, substantial perioperative blood loss has been associated with systemic complications, especially in elderly individuals [1, 2]. Many methods have been employed to attempt to manage blood loss including tourniquet application, blood transfusion, administration of haemostatic agents and autologous donation [3]. Allogenic blood transfusion may increase the risk of adverse events, such as virus infections, immunologically mediated diseases and cardiovascular dysfunction, potentially resulting in life-threatening effects on patients and an added financial burden on the patients [4].

Recently, the use of tranexamic acid (TXA) in total knee and hip arthroplasty has become popular among the orthopaedic surgeons. TXA is a synthetic analogue of an amino acid with biological activity which can inhibit plasminogen from dissolving clots [5]. In previous studies, combined intravenous and topical applications of TXA have been reported to be associated with reduced perioperative blood loss and transfusion units [6]. Despite this research, the lack of large published studies and small sample sizes makes it unclear whether the combined application of TXA is superior to single intravenous application. Therefore, we performed a meta-analysis to evaluate the efficiency and safety of combined intravenous and topical methods of application versus single intravenous of tranexamic acid in primary total knee and hip arthroplasty.

## Methods

### Search strategy

Studies were considered for inclusion if they met the following criteria: (1) published randomised control trials (RCTs); (2) included a patient population that underwent total knee or hip arthroplasty, with an experimental group that received combined intravenous and topical application of TXA and a control group that received a single application of TXA; (3) reported surgical outcomes including haemoglobin decline or postoperative haemoglobin level, total blood loss, drainage volume, transfusion requirements, length of stay and operation time as well as surgery-related adverse effects, such as wound infection, deep vein thrombosis (DVT) and pulmonary embolism (PE). Studies were excluded from the present meta-analysis if they included incomplete data. All analyses were based on previous published studies; thus, no ethical approval and patient consent are required.

**Fig. 1:**
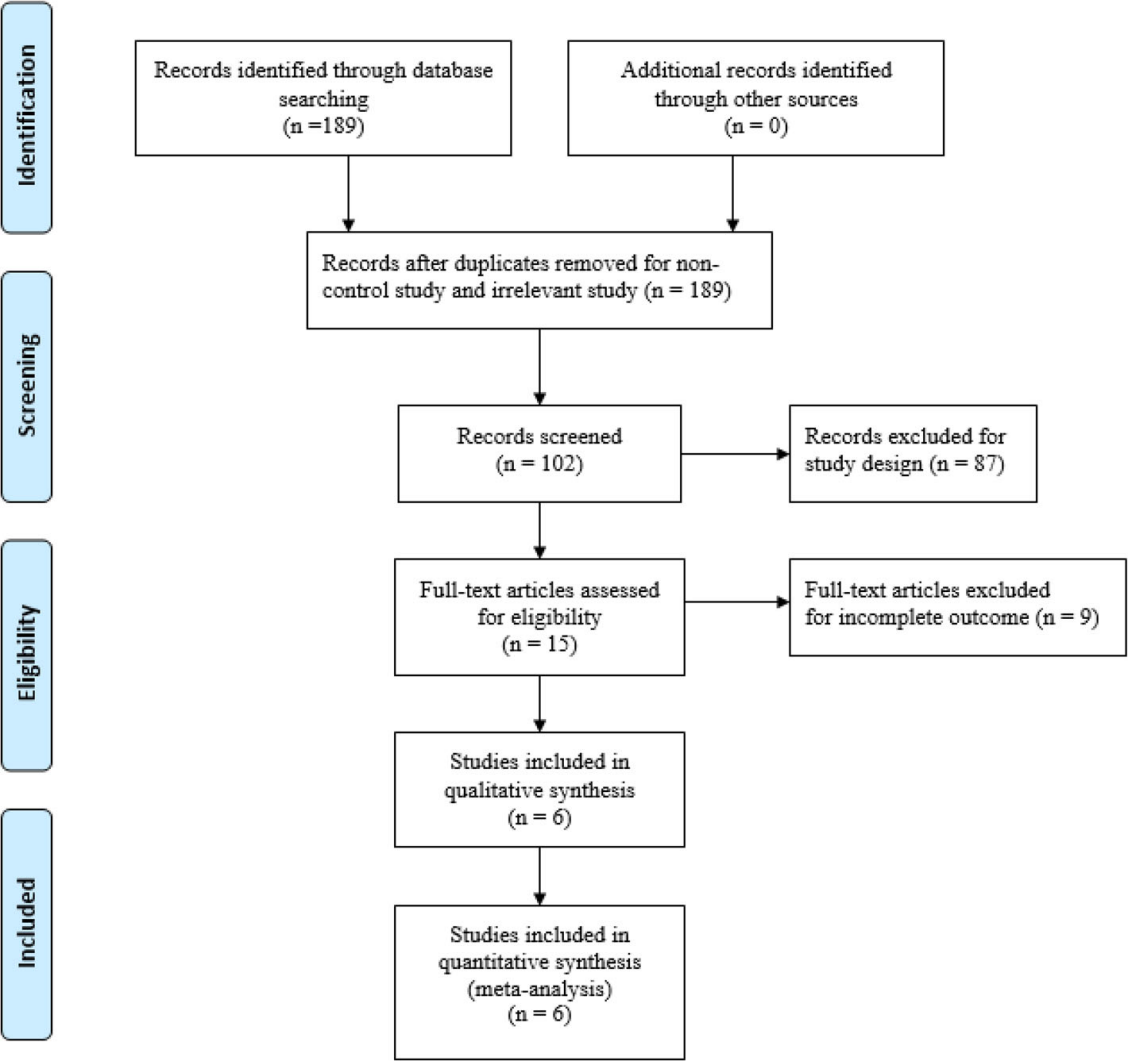
Search results and the selection procedure

Potentially relevant studies were identified from electronic databases including MEDLINE (from 1966 to 25 September 2016), PubMed (from 1966 to 25 September 2016), Embase (from 1980 to 25 September 2016), ScienceDirect (from 1985 to 25 September 2016) and the Cochrane Library. The following keywords were used on combination with Boolean operators AND or OR: “total knee replacement OR arthroplasty”, “total hip replacement OR arthroplasty” and “tranexamic acid”, “blood loss” or “blood transfusion”. The bibliographies of retrieved trials and other relevant publications were cross-referenced to identify additional articles. The search process was performed as presented in Fig. 1.

### Date extraction

Two reviewers independently scanned the abstracts of the potentially included studies. Subsequently, the full text of the studies that met the inclusion criteria was screened, and a final decision was made. Disagreement was resolved by consulting a senior reviewer. Two of the authors independently extracted data from the included studies. Corresponding authors were consulted for details if the data were incomplete. The following data were extracted and recorded in a spreadsheet: first author names, publication year, baseline characteristics, intervention procedures, sample size, transfusion trigger and outcome parameters. Other relevant data were also extracted from individual studies. Primary outcomes were total blood, haemoglobin decline and transfusion requirements. Secondary outcomes were length of stay, operation time and TXA-related adverse effects, such as superficial infection, DVT or PE.

### Quality assessment

A quality assessment of each randomised trial was performed by two reviewers based on the Cochrane Handbook for Systematic Reviews of Interventions. Disagreement was resolved by consulting a senior reviewer. We created a “risk of bias” table that included the following elements: random sequence generation, allocation concealment, blinding, incomplete outcome data, free of selective reporting and other bias.

### Data analysis and statistical methods

Pooling of data was carried out using RevMan 5.1 (The Cochrane Collaboration, Oxford, UK). Statistical heterogeneity was assessed based on the value of *P* and *I* ^2^ values and using standard chi-square tests. When *I* ^2^ ≥ 50% or *P* < 0.1, significant heterogeneity was indicated and a random-effects model was applied for meta-analysis. Otherwise, a fixed-effects model was used. Sensitivity analysis is conducted to find the source if possible under significant heterogeneity. Dichotomous outcomes were expressed as risk differences (RDs) with 95% confidence intervals (CIs). For continuous outcomes, mean differences (MDs) and 95% confidence intervals (CIs) were calculated.

## Results

### Search result

A total of 189 studies were identified through the initial search. By scanning the abstracts, 183 reports that did not meet inclusion criteria were excluded from the meta-analysis and no grey studies were included. Finally, six randomised control trials (RCTs) [7–12] that had been published between 2014 and 2016 were used for this meta-analysis. These studies included 343 patients in experimental groups and 344 patients in control groups. The experimental groups received combined intravenous and topical TXA, while control groups received single intravenous TXA. Sample sizes ranged from 60–184 patients. Doses of TXA in each study are summarised in Table 1 as well as the demographic characteristics of the participants. The primary outcomes were total blood loss, haemoglobin decline, transfusion requirement and drainage volume. The secondary outcomes were operation time, length of stay and adverse side effects. The duration of follow-up ranged from 1 month to 2 years.

**Table 1:** Cohort characteristics

Studies	Cases	Mean age	Female patient	Surgical methods	TXA intervention	Prophylactic antithrombotic	Transfusion trigger follow-up	
	(E/C)	(E/C)	(E/C)					
Huang 2014 [7]	92/92	65.4/64.7	55/62	TKA	E:1.5 g topical injection +1.5 g i.v.	LMWH, 6000 IU	HB less than 7 g/dL	1–3 months
					C:3 g i.v.			
Nielsen 2016 [8]	30/30	65.5/63.2	17/15	TKA	E:3 g topical injection + 1 g i.v.	Rivaroxaban, 10 mg	HB less than 7.5 g/dL	3 months
					C:3 g i.v.			
Jain 2016 [12]	59/60	68.3/70.0	39/36	TKA	E:pre-op.15 mg/kg i.v. + post-op.10 mg/kg i.v. + 2 g topical injection	Aspirin, 75 mg	HB less than 7 g/dL	NS
					C:pre-op.15 mg/kg i.v. + post-op.10 mg/kg i.v			
Xie 2016 [11]	70/70	60.5/59.5	48/50	THA	E:1 g topical injection + 2 g i.v.	Enoxaparin, 6000 IU	HB less than 7 g/dL	1–3 months
					C:1.5 g i.v.			
Wu 2016 [10]	42/42	60.1/59.5	19/21	Reversion THA	E:3 g topical injection +15 mg/kg i.v.	LMWH, 6000 IU	HB less than 8 g/dL	3 months
					C:15 mg/kg i.v.			
Song 2016 [9]	50/50	70.8/69.2	43/44	TKA	E: 1.5 g topical injection +10 mg/kg i.v. C:E:pre-op.10 mg/kg i.v. + post-op.10 mg/kg	Not performed	HB less than 8 g/dL	1 month

*E* experiment group, *C* control group, *i.v.* intravenous injection, *LMWH* low-molecular-weight heparin, *HB* haemoglobin, *pre-op* preoperative, *post-op* postoperative, *NS* not stated

### Risk of bias assessment

The Cochrane Handbook for Systematic Review of Interventions was consulted to assess the quality of the trials. All RCTs provided clear inclusion and exclusion criteria and described their randomization methodology, and three studies [8, 9, 12] described the use of computer-generated randomization. Five studies [7–11] reported allocation concealment by closed envelope or other techniques. Double blinding was reported in five of the trials [7–10, 12]; however, only one study [8] had attempted to blind assessors. Intention-to-treat analysis was not performed in any of the RCTs; therefore, there was a potential risk of type II statistical errors occurring. No unclear bias was identified, due to incomplete outcome data or selective outcome reporting. The methodological quality assessment is summarised in Table 2. Each risk of bias item is presented as a percentage across all the included studies, which indicates the proportion of the different levels of risk of bias for each item (Table 3).

**Table 2:**
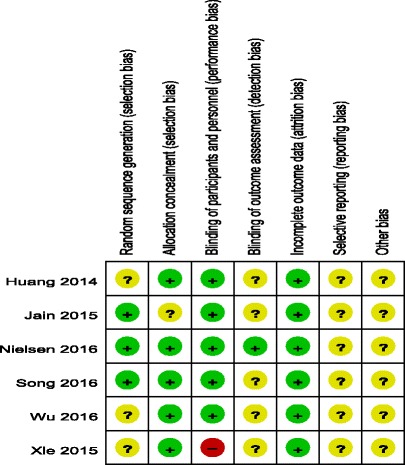
Methodological quality of the randomized controlled trials

**Table 3:**
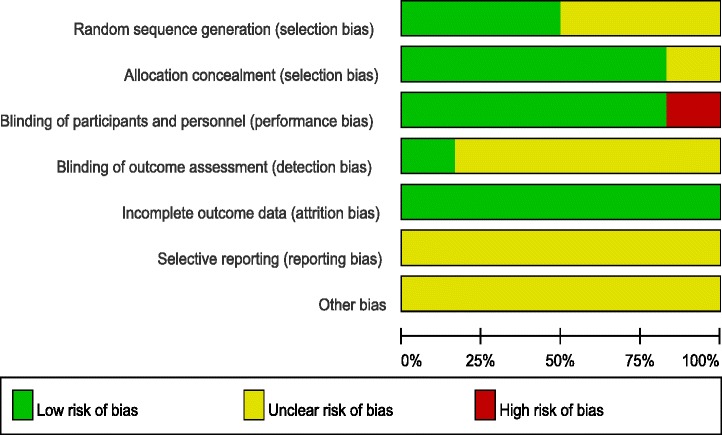
Risk of bias

### Outcomes for meta-analysis

#### Total blood loss

**Fig. 2:**
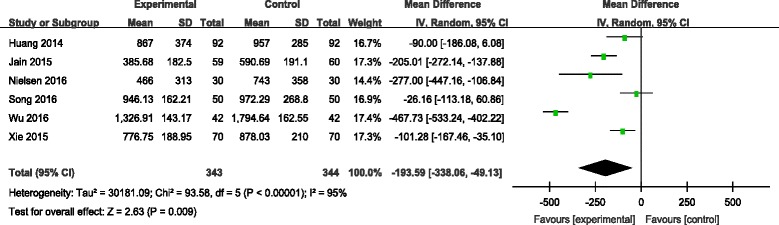
Forest plot diagram showing effect of combination TXA on total blood loss

Six articles [7–12] reported the outcomes of total blood loss following operation. There was significant heterogeneity among the studies (*χ* ^2^ = 93.58, df = 5, *I* ^2^ = 95%, *P* < 0.00001); therefore, a random-effects model was used. Pooled results demonstrated that total blood loss in control groups was significantly higher than in experimental groups (MD = −193.59, 95% CI −338.06 to −49.13, *P* = 0.009; Fig. 2).

#### Transfusion rate

**Fig. 3:**
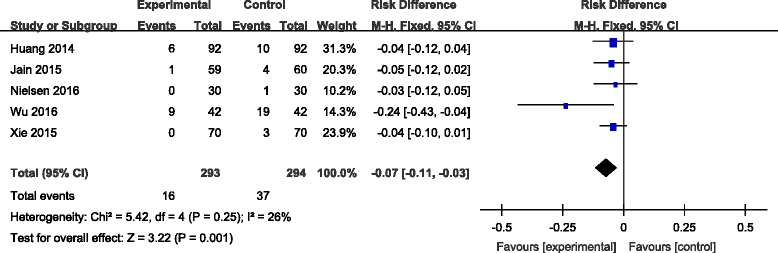
Forest plot diagram showing effect of combination TXA on transfusion rate

Transfusion rates were reported in five studies [7, 8, 10–12]. No significant heterogeneity among these studies was found; therefore, a fixed-effects model was applied (*χ* ^2^ = 5.42, df = 4, *I* ^2^ = 26%, *P* = 0.25). There was a significant difference between the two groups in transfusion rate (RD = −0.07, 95% CI −0.12 to −0.03, *P* = 0.001; Fig. 3).

#### Haemoglobin decline

**Fig. 4:**
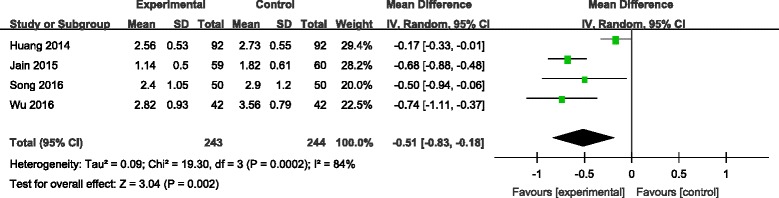
Forest plot diagram showing effect of combination TXA on haemoglobin decline

Four studies [7, 9, 10, 12] reported the outcomes of haemoglobin decline following operation. There was significant heterogeneity among these studies (*χ* ^2^ = 19.30, df = 3, *I* ^2^ = 84%, *P* = 0.0002); therefore, a random-effects model was used. Pooled results demonstrated that haemoglobin decline in control groups was significantly higher than in experimental groups (MD = −0.51, 95% CI −0.83 to −0.18, *P* = 0.01; Fig. 4).

#### Drainage volume

**Fig. 5:**
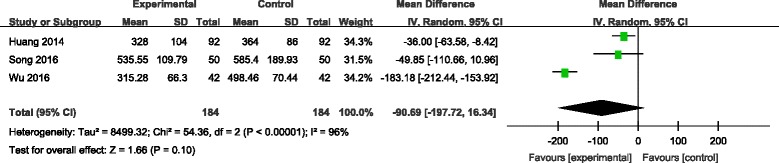
Forest plot diagram showing effect of combination TXA on drainage volume

Drainage volume was provided in three studies [7, 9, 10]. Significant heterogeneity among these studies was found; therefore, a random-effects model was used (*χ* ^2^ = 54.36, df = 2, *I* ^2^ = 96%, *P* < 0.00001). There was no significance between the two groups in drainage volume (MD = −90.69, 95% CI −197.72 to 16.34, *P* = 0.10; Fig. 5).

#### Operation time

**Fig. 6:**
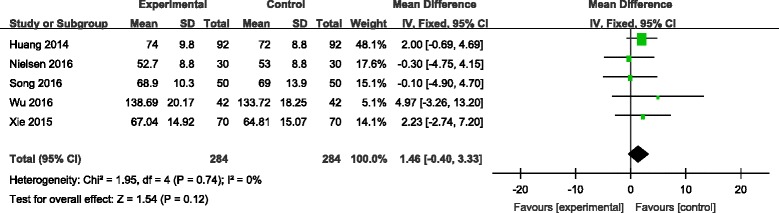
Forest plot diagram showing effect of combination TXA on operation time

Operation time was reported in five studies [7–11]. There was no significant heterogeneity among the pooled data; therefore, a fixed-effects model was used (*χ* ^2^ = 1.95, df = 4, *I* ^2^ = 0%, *P* = 0.74). There was no significance between the two groups in operation time. (MD = 1.46, 95% CI −0.40 to 3.33, *P* = 0.12; Fig. 6).

#### Length of hospital stay

**Fig. 7:**
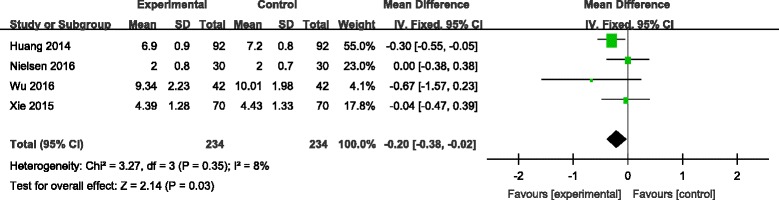
Forest plot diagram showing effect of combination TXA on length of stay

Four studies reported the length of hospital stay between groups [7, 8, 10, 11]. No significant heterogeneity was identified in the pooled results; therefore, a fixed-effects model was used (*χ* ^2^ = 3.27, df = 2, *I* ^2^ = 8%, *P* = 0.35). There was no significant difference between the two groups in length of hospital stay (LOS). (MD = −0.20, 95% CI −0.38 to −0.02, *P* = 0.03; Fig. 7).

#### Superficial infection

**Fig. 8:**
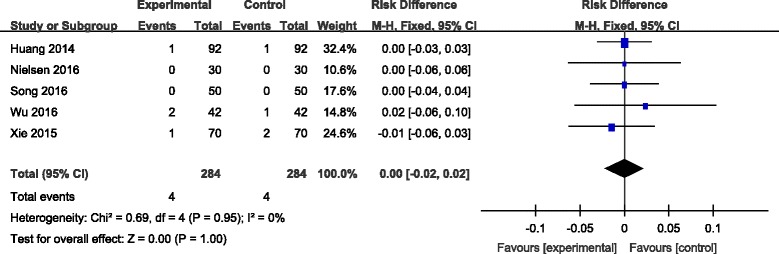
Forest plot diagram showing effect of combination TXA on risk of superficial infection

Superficial infection incidence was reported in five studies [7–11]. No significant heterogeneity among these studies was found; therefore, a fixed-effects model was used (*χ* ^2^ = 0.69, df = 4, *I* ^2^ = 0%, *P* = 0.95). There was no significant difference between the two groups in the incidence of superficial infection (RD = 0.00, 95% CI −0.02 to 0.02, *P* = 1.00; Fig. 8).

#### Deep vein thrombosis

**Fig. 9:**
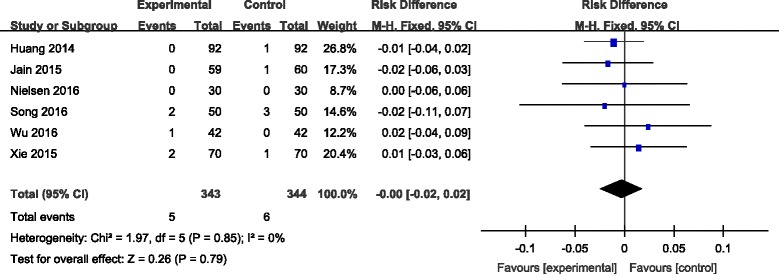
Forest plot diagram showing effect of combination TXA on risk of deep vein thrombosis

Six articles [7–12] reported the incidence of DVT following joint replacement. A fixed-effects model was used due to the low-significant heterogeneity among these studies (*χ* ^2^ = 1.97, df = 5, *I* ^2^ = 0%, *P* = 0.85). No significant difference was found between the groups (RD = −0.00, 95% CI −0.02 to 0.02, *P* = 0.79; Fig. 9).

## Discussion

Substantial studies have assessed the efficiency and safety of TXA by various routines in total knee and hip arthroplasty. However, intravenous combined topical use of TXA was seldom studies. Therefore, this is the first meta-analysis to evaluate the efficiency and safety of combined use of intravenous and topical versus single intravenous TXA in primary total knee and hip arthroplasty. The most important finding of the meta-analysis was that combined application of intravenous and topical tranexamic acid in patients with TKA and THA was associated with significantly reduced total blood loss, postoperative haemoglobin decline and transfusion requirements compared to single intravenous application where no increased risk of the incidence of infection, DVT or PE was identified. More interestingly, our finding indicated that there is a shortened length of stay in combined groups.

Although the overall methodological quality of the six selected studies was relatively high, some methodological weaknesses existed which would have affected their results. For example, the sample size of all studies was relatively small and the statistical efficacy could be improved by including more studies with larger sample sizes as well as long-term follow-ups with patients. Three studies did not clarify their randomization methods, and one study did not indicate allocation concealment. We attempted to find the source of heterogeneity for total blood loss; however, it still existed. Publication bias of the meta-analysis also influenced results. These shortcomings should be taken into consideration when analysing the results of this meta-analysis.

**Fig. 10:**
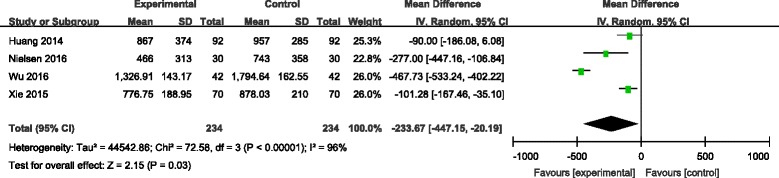
Sensitivity analysis on total blood loss

TXA is an anti-fibrinolytic agent that inhibits fibrinolysis by reversibly blocking the lysine-binding sites of plasminogen. This method is commonly used in orthopaedic surgery to decrease intraoperative blood loss and transfusion amounts. TXA can be applied by various routes including intravenous, intraarticular, oral and intramuscular. For patients undergoing TKA, the best suitable method for rapidly increasing and maintaining the therapeutic concentration of TXA is by the intravenous route. Topical application of TXA has many conceptual and realistic advantages. Firstly, TXA inhibits local activation of fibrinolysis as well as systemic activation from local mediators after tourniquet release [13]. Additionally, a higher concentration of local TXA in the knee joint should result in greater thrombus formation and a lower time to vascular occlusion [14]. Further, topical TXA has very low systemic absorption. This meta-analysis indicated that combined application of TXA was associated with significantly reduced total blood loss compared with a single application of TXA. Because there was significant heterogeneity among the studies, we performed sensitivity analysis to excluded studies whose sample size was small. However, significant heterogeneity still existed (Fig. 10).

Substantial numbers of previous studies have reported total blood loss in patients undergoing TKA and THA; for instance, TKA without antifibrinolytics was associated with blood loss ranging from 761 to 1784 ml [15–18] and 7.7 to 18.93% [19–22] of these patients needed transfusion to relieve anaemia. However, transfusion was considered undesirable due to its associated risks with adverse reactions. It has been frequently identified that when either systemic or topical administration of TXA was administrated, transfusion was required less frequently. The current meta-analysis showed that combined application of TXA was associated with a further significant reduction in transfusion requirements and haemoglobin decline compared to single application in patients with total knee and hip replacement.

Long periods of bedridden patients and extensive operation times increase the expenses associated with operations. More importantly, adverse events, such as, hypostatic pneumonia, deep vein thrombosis and pulmonary embolism are associated with increased morbidity and mortality [19]. Early weight-bearing and rehabilitation have been shown to contribute to better functional outcomes following knee and hip arthroplasty. This meta-analysis indicated that combined administration of TXA was associated with shortened bedridden durations following total knee and hip arthroplasty compared to single application. We speculate that the results may be caused by reduced blood loss and transfusion requirements which are another important finding.

Infection is relatively rare after TKA but can be devastating in terms of morbidity and associated costs. This meta-analysis showed that there was no significant difference in the incidence of infection, which was 4/284 in the experimental groups and 4/284 in the control groups. The overall infection incidence was 1.4%, which is in accordance with previous studies which reported incidences ranging between 1 and 3% [23]. High-quality trials with larger sample sizes are required to further explore the correlation between infection and application of TXA.

DVT has been identified as a common complication that may develop into PE and even result in death following major orthopaedic surgery. Most participants received routine prophylactic antithrombotic therapy. Previous studies have reported that there was a higher risk of developing DVT and PE when TXA was utilised [24]. Perhaps this finding occurred because TXA, an antifibrinolytic agent, has the tendency to increase the risk of clotting. Therefore, there was a possibility that combined application of TXA would be more likely to result in the formation of a thrombus. However, no significant difference was found between the groups in terms of the incidence of DVT or PE. As identified for the other factors, further large-scale trials are needed to confirm the observed outcomes.

Several potential limitations that should be noted: (1) Only six RCTs were included, and the sample size was relatively small. (2) Some outcome parameters such as the visual pain score (VAS) and range of motion (ROM) were not fully described and could not be included in the meta-analysis. (3) Because of the limited number of included studies, subgroup analyses were not performed for some outcomes; therefore, we could not determine the source of heterogeneity. (4) Short-term follow-up may lead to underestimation of complications. (5) Publication bias is an inherent weakness that exists in all meta-analyses.

## Conclusions

Combined administration of TXA in patients with total knee and hip arthroplasty was associated with significantly reduced total blood loss, transfusion requirements, postoperative haemoglobin decline and length of stay compared to single application alone but was not associated with prolonged operation time. Moreover, no adverse effects, such as superficial infection, DVT or PE, were associated with TXA. We suggest that combined administration of TXA demonstrated excellent clinical efficacy and safety in patients with total knee and hip arthroplasty. More importantly, well-designed studies with larger sample size are needed to provide further reliable evidence for the combined use of TXA.

## Abbreviations

DVT: Deep vein thrombus
KA: Total knee arthroplasty
PE: Pulmonary embolism
RCT: Randomized controlled trials
THA: Total hip arthroplasty
TXA: Tranexamic acid
